# An Exploratory Transcriptomic Classification Model for Psoriasis Based on Apoptosis-Associated and Proliferation–Apoptosis-Coupled Genes Using Explainable Machine Learning

**DOI:** 10.3390/ijms27125441

**Published:** 2026-06-16

**Authors:** Xinhao Liu, Wenqing Fu, Jiachen Li, Mengyang Jing, Xuli Zhu, Wenhao Bo

**Affiliations:** 1 Center for Computational Biology, College of Biological Sciences and Technology, Beijing Forestry University, Beijing 100083, China; liuxinhao@bjfu.edu.cn (X.L.); ljwyjljc1003jjj@outlook.com (J.L.); decarac1e@bjfu.edu.cn (M.J.); 2 State Key Laboratory of Tree Genetics and Breeding, National Engineering Research Center of Tree Breeding and Ecological Restoration, Key Laboratory of Genetics and Breeding in Forest Trees and Ornamental Plants, Ministry of Education, College of Biological Sciences and Biotechnology, Beijing Forestry University, Beijing 100083, China; wenqingfu@bjfu.edu.cn

**Keywords:** psoriasis, apoptosis, explainable machine learning, gradient boosting machine, transcriptomic classification

## Abstract

This study aimed to integrate apoptosis-associated and proliferation–apoptosis-coupled transcriptomic signatures with explainable machine learning to construct an exploratory molecular classification model for psoriasis. Transcriptomic datasets GSE30999 and GSE53552 were merged as the skin-tissue training cohort, and GSE55201, a whole-blood transcriptomic dataset, was used as an independent cross-tissue external validation cohort. Differential expression analysis identified 3707 DEGs, and intersection with GeneCards apoptosis-related genes yielded 894 overlapping genes. After PPI-based hub gene selection, eight machine learning algorithms were exploratorily compared within a preselected 25-gene feature space. DALEX-based permutation feature importance analysis identified a five-gene apoptosis-associated and proliferation–apoptosis-coupled signature comprising *CCNB1*, *KIF11*, *HDAC1*, *TPX2*, and *MELK*. The five-gene model achieved an AUC of 0.966 in the training cohort and 0.811 in the external whole-blood validation cohort, indicating moderate cross-tissue generalizability. Calibration and decision-curve analyses were performed only in the training cohort and should be interpreted as exploratory analyses rather than evidence of clinical utility. Overall, this study provides an interpretable transcriptomic classification framework for distinguishing psoriasis from healthy controls, while its ability to differentiate psoriasis from clinically similar dermatoses remains to be validated in independent disease-control cohorts.

## 1. Introduction

Psoriasis is a chronic relapsing immune-mediated inflammatory skin disease with a global prevalence of approximately 2–3%, substantially compromising patients’ quality of life [1,2,3,4]. Its core pathological features include keratinocyte hyperproliferation, parakeratosis, and inflammatory cell infiltration in the dermis. Although biologic agents targeting interleukin (IL)-17 and IL-23 have markedly improved therapeutic outcomes, a subset of patients still experience treatment failure, loss of response, or relapse after discontinuation due to disease heterogeneity [5,6].

Among the various pathological mechanisms implicated in psoriasis, dysregulation of apoptosis has been increasingly recognized as a key driving factor [7,8,9]. Under physiological conditions, epidermal keratinocytes undergo an orderly cycle of proliferation, differentiation, and apoptosis to maintain skin barrier integrity [10]. In psoriatic lesions, however, keratinocytes exhibit pronounced apoptosis resistance, including downregulation of the Fas/FasL pathway, overexpression of anti-apoptotic Bcl-2 family proteins, and suppression of the caspase cascade [11,12,13], resulting in a proliferation–apoptosis imbalance that forms the cellular basis of psoriatic plaque formation. Dysregulated apoptosis in psoriatic keratinocytes is not merely a passive consequence of inflammation but a central pathogenic feature: resistance to programmed cell death allows the persistence of hyperproliferative clones and contributes to the characteristic acanthosis and parakeratosis of psoriatic plaques [14]. Clinically, the diagnosis of psoriasis still relies primarily on morphological observation of skin lesions and histopathological biopsy. The former is highly dependent on physician experience and has limited discriminative power for early, atypical, or overlapping lesions resembling eczema or seborrheic dermatitis. Although histopathological biopsy is considered the diagnostic gold standard, it is invasive, time-consuming, and subject to sampling-site variability, making it unsuitable for large-scale early screening. Recent deep learning models based on skin lesion image recognition have achieved progress in automated psoriasis identification [15,16]; however, they rely fundamentally on macroscopic morphological features, are unable to reveal molecular-level abnormalities, and show limited efficacy in mild or subclinical lesions. Therefore, developing objective and molecularly grounded biomarkers is of considerable significance for improving the molecular characterization of psoriasis. However, whether such biomarkers can support differential diagnosis against clinically similar dermatoses requires dedicated validation in disease-control cohorts.

With the rapid development of high-throughput genomic technologies and public repositories such as the Gene Expression Omnibus (GEO), mining disease-related biomarkers from large-scale transcriptomic data using bioinformatics approaches has become a major research paradigm [17,18,19]. Machine learning algorithms, owing to their inherent advantages in processing high-dimensional nonlinear data, have been widely applied to the construction of disease diagnostic models [20,21,22,23]. However, traditional single-algorithm approaches face two critical challenges in clinical translation. First, different algorithms may yield substantially divergent feature selection results due to their distinct mathematical assumptions and optimization objectives, undermining the robustness of findings derived from any single algorithm [20,21,22,23]. Second, high-performance models such as Gradient Boosting Machine (GBM) and neural networks are inherently “black-box” models whose complex internal decision logic is difficult to interpret, thereby limiting their adoption in clinical practice [24,25].

To systematically address the aforementioned interpretability bottleneck, explainable machine learning has gained increasing attention in biomedical research in recent years [26,27]. Current mainstream explainability methods include SHapley Additive exPlanations (SHAP), Local Interpretable Model-agnostic Explanations (LIME), and Descriptive Machine Learning EXplanations (DALEX), each with distinct theoretical foundations and application scenarios. SHAP, derived from Shapley value theory in cooperative game theory, computes the marginal contribution of each feature across all possible feature combinations to achieve fair attribution for model predictions; it supports both global feature importance ranking and local sample-level interpretation but suffers from exponentially increasing computational complexity with the number of features, rendering it costly for high-dimensional gene expression data [27]. LIME adopts a local approximation strategy, generating perturbed samples around a target instance and fitting a simple linear surrogate model to approximate the local decision boundary of the black-box model; although computationally efficient and model-agnostic, its local explanations depend on the perturbation sampling process and may yield inconsistent results across different runs, resulting in insufficient stability [27,28]. In contrast, DALEX employs a permutation-based feature importance algorithm that randomly shuffles each variable individually and observes the resulting drop in model performance to globally quantify each variable’s marginal contribution. This method is independent of model internals and produces stable, reproducible results [29].

**Figure 1:**
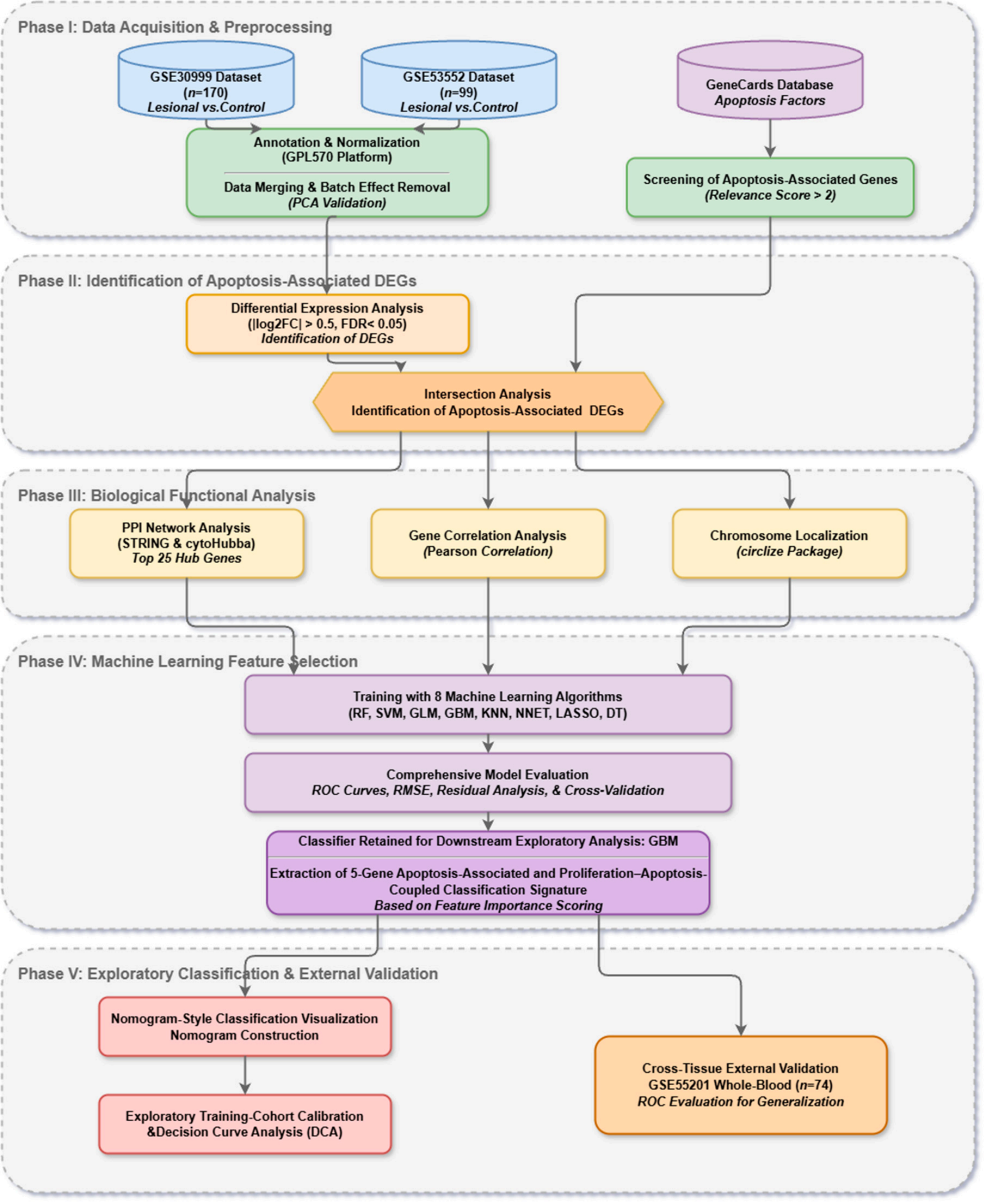
Technical route of this study. The solid arrows indicate the direction of the analytical workflow.

Based on the above background, this study explores an integrated molecular classification strategy for psoriasis that combines apoptosis-associated genes with explainable machine learning algorithms. Specifically, multiple psoriasis transcriptomic datasets from the GEO database were integrated and intersected with the GeneCards apoptosis-related gene set, followed by hub gene selection through PPI network analysis. Eight mainstream machine-learning algorithms were then exploratorily compared within the preselected feature space, and bootstrap 95% confidence intervals were calculated for cross-validation AUCs to quantify performance uncertainty. The DALEX framework was subsequently used to extract core feature genes, ultimately constructing a nomogram-style visualization of the five-gene classification model and evaluating it in an independent external cohort. The intended application of the proposed model is to explore apoptosis-related transcriptomic signatures that distinguish psoriasis from healthy controls at the transcriptomic level and to provide a research-oriented molecular stratification framework for psoriasis. Although such molecular signatures may have potential relevance for future differential-diagnostic studies, the present validation design does not directly test discrimination between psoriasis and clinically similar skin diseases. Therefore, the model should not be interpreted as a validated tool for differentiating psoriasis from eczema, seborrheic dermatitis, lichen planus, or cutaneous T-cell lymphoma. The main methodological features of this study are reflected in three aspects. First, biology-driven gene screening was performed starting from the core pathological feature of psoriasis, namely the imbalance between apoptosis and proliferation, ensuring that the identified feature genes possess both statistical significance and pathophysiological relevance. Second, a horizontal comparison strategy encompassing eight mainstream machine learning algorithms was employed, with the Area Under the Receiver Operating Characteristic Curve (AUC), bootstrap 95% confidence intervals, standard classification metrics (Accuracy, Precision, Recall, F1-score), and Root Mean Square Error (RMSE)-based stability serving as exploratory evaluation metrics on a preselected 25-gene feature set. Because upstream feature selection was performed before cross-validation, this comparison was not intended to provide an unbiased nested estimate of algorithmic superiority, but rather to guide exploratory downstream model construction and reduce reliance on a single arbitrary classifier. Third, the DALEX explainability framework was introduced to quantitatively interpret the relative contribution of each feature gene to diagnostic prediction, enhancing the interpretability of the model. The overall study design, including data preprocessing, apoptosis-related gene screening, machine learning model construction, explainability analysis, and external validation, is summarized in Figure 1.

## 2. Results

### 2.1. Data Integration, Differential Expression Analysis, and Apoptosis-Related Gene Screening

**Figure 2:**
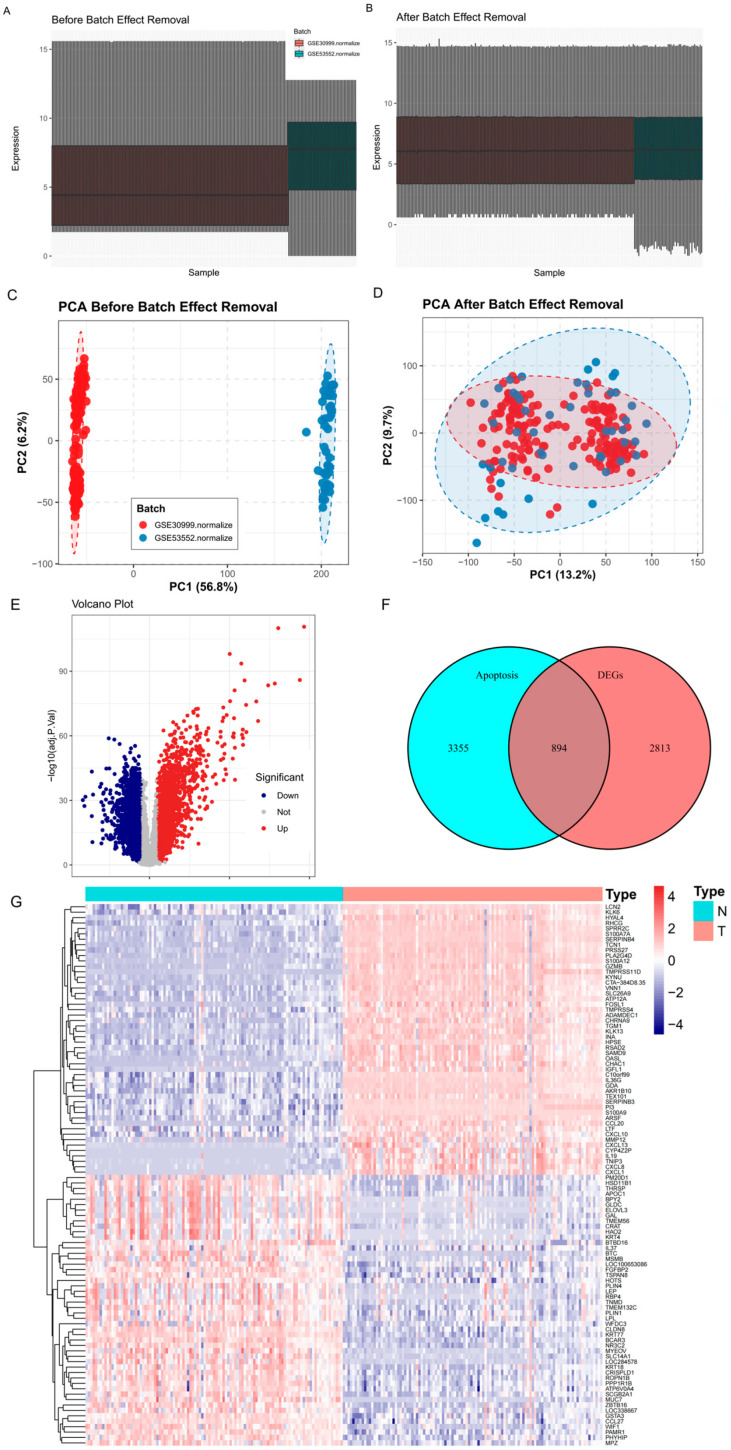
Data integration, differential expression analysis, and apoptosis-related gene screening. (**A**) Boxplot of gene expression distribution before batch effect removal. (**B**) Boxplot after batch effect correction. (**C**) PCA plot before correction, showing clear separation between datasets. (**D**) PCA plot after correction, with samples uniformly intermixed. The red and blue dashed ellipses represent the 95% confidence regions for the respective groups. (**E**) Volcano plot of DEGs. (**F**) Venn diagram showing the intersection of DEGs with apoptosis-related genes, yielding 894 overlapping genes. (**G**) Hierarchical clustering heatmap of the top 100 DEGs. N: normal controls; T: psoriasis.

After merging GSE30999 and GSE53552, batch effect correction was performed using the removeBatchEffect function of the limma package (version 3.66.0). PCA revealed clear separation of samples from the two datasets in principal component space prior to correction (Figure 2A,C), indicating a substantial batch effect. After correction, samples were uniformly intermixed (Figure 2B,D), confirming that the batch effect had been effectively eliminated.

Differential expression analysis on the integrated training cohort identified 3707 significantly differentially expressed genes using the criteria |log2FC| > 0.5 and FDR < 0.05. The volcano plot (Figure 2E) displays the distribution of upregulated and downregulated genes, revealing extensive gene expression reprogramming in psoriatic lesions. The hierarchical clustering heatmap (Figure 2G) confirmed that these DEGs exhibited markedly distinct expression profiles between psoriatic and control samples, with clear separation under unsupervised clustering. A total of 4249 apoptosis-related genes with relevance scores >2 were retrieved from the GeneCards database. Venn diagram analysis identified 894 overlapping genes between the 3707 DEGs and the 4249 apoptosis-related genes (Figure 2F), which were defined as apoptosis-related DEGs.

### 2.2. PPI Network Analysis, Hub Gene Identification, Co-Expression Relationships, and Chromosomal Distribution

**Figure 3:**
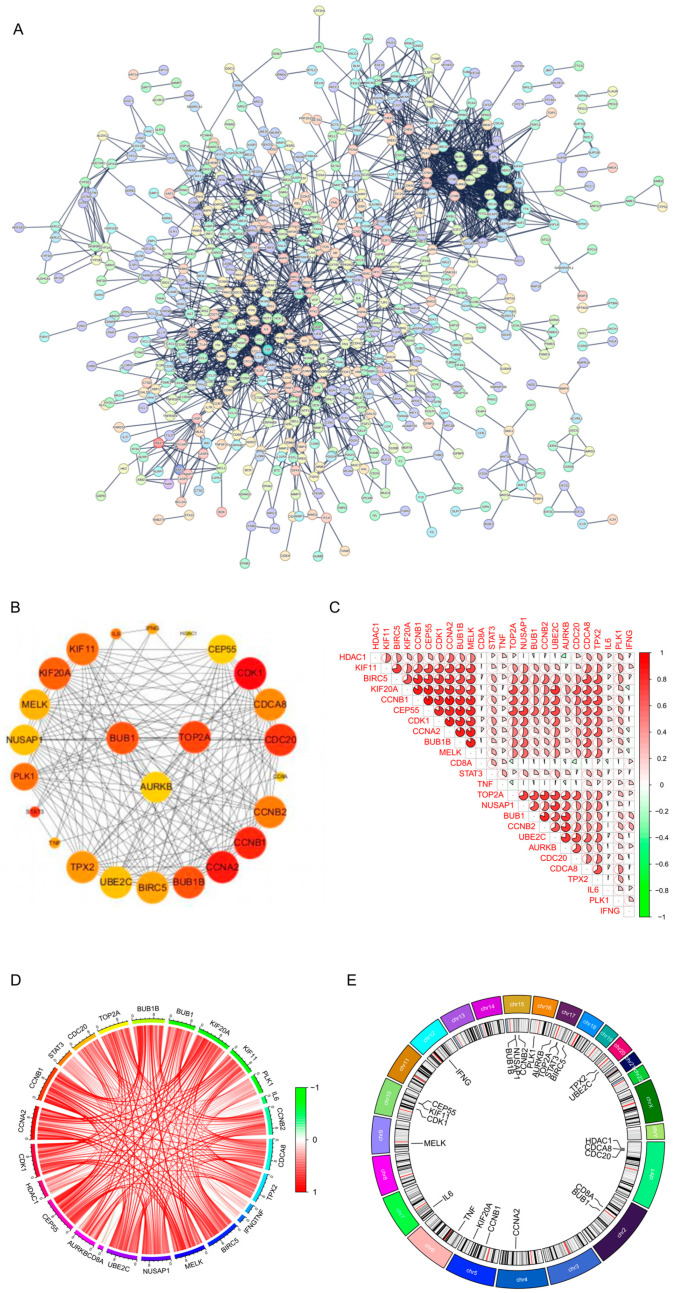
PPI network construction, hub gene identification, and co-expression and chromosomal distribution. (**A**) PPI network of 894 apoptosis-related DEGs (STRING, confidence = 0.9). (**B**) Subnetwork of the 25 core hub genes identified by cytoHubba (v0.1). Node color from yellow to red indicates increasing connectivity; node size is proportional to degree. (**C**) Pearson correlation heatmap of the 25 hub genes. (**D**) Chord diagram of the co-expression network. (**E**) Circos plot of hub gene chromosomal distribution (hg38 assembly).

The 894 apoptosis-related DEGs were submitted to the STRING database (species: Homo sapiens; confidence threshold = 0.9) to construct a PPI network, yielding a network comprising 881 nodes and 2099 edges (Figure 3A), with a PPI enrichment *p*-value < 1.0 × 10^−16^. This indicates that the encoded proteins exhibit significantly more interactions than expected by chance, validating the biological coherence of the selected gene set. Using the cytoHubba plugin with a combination of Degree, MCC, and Closeness topological algorithms, the top 25 core hub genes were identified based on Degree ranking (Figure 3B). These genes occupy highly connected central positions within the network, suggesting that they may play critical regulatory roles in the apoptosis-related mechanisms underlying psoriasis.

To further characterize the transcriptional coordination among hub genes, Pearson correlation analysis was performed on the 25 genes. The correlation heatmap (Figure 3C) and chord diagram (Figure 3D) revealed widespread and predominantly positive correlations among the majority of hub genes, suggesting functional synergy at the transcriptional level and possibly shared upstream regulatory drivers. A Circos chromosomal plot based on the hg38 assembly (Figure 3E) mapped the 25 hub genes to their specific physical locations on human chromosomes. Notably, *HDAC1* and *TPX2* are both located on chromosome 15, and *CCNB2* and *AURKB* colocalize on chromosome 17. Such chromosomal proximity suggests the possibility of shared cis-regulatory elements or coordinated epigenetic regulation among spatially clustered hub genes.

### 2.3. Exploratory Classifier Comparison Within the Preselected 25 Hub-Gene Feature Set

To exploratorily compare classifier performance on the preselected 25 hub-gene feature set, expression data of these genes were input into eight machine learning algorithms—RF, SVM, GLM, GBM, KNN, NNET, LASSO, and DT—for model training. Their performance was evaluated in terms of discriminatory ability, standard classification metrics, and predictive stability. Because the upstream feature selection steps had been performed on the full training cohort before cross-validation, these results should be interpreted as descriptive comparisons within a fixed feature set rather than unbiased nested estimates of algorithmic superiority. Therefore, this analysis compares classifier behavior under a constrained and shared feature space, rather than comparing fully independent end-to-end modeling pipelines with algorithm-specific feature-selection procedures.

Cross-validation ROC analysis showed that all eight classifiers achieved relatively high discrimination within the preselected 25-gene feature set. KNN showed the highest point-estimate AUC of 0.978, followed by RF (AUC = 0.974), SVM (AUC = 0.971), LASSO (AUC = 0.969), NNET (AUC = 0.962), GBM (AUC = 0.962), GLM (AUC = 0.933), and DT (AUC = 0.890). These AUC values should therefore be interpreted as performance estimates of different classifiers using the same 25 input genes, not as evidence that one complete feature-selection-and-classification pipeline is superior to another. Importantly, because DEG screening, apoptosis-gene intersection, PPI-based hub-gene identification, and feature preselection were performed before cross-validation, these AUC values are likely optimistically biased and should be interpreted as exploratory comparisons among algorithms under a fixed preselected feature set, rather than unbiased estimates of out-of-sample generalization. Standard classification metrics at the default 0.5 probability threshold—including Accuracy, Precision, Recall, and F1-score—were further computed to provide a descriptive assessment of classification performance within the preselected feature set. To provide a more intuitive overview of performance trade-offs and uncertainty among the eight classifiers, the cross-validation AUCs, bootstrap 95% confidence intervals, and standard classification metrics are summarized in Table 1. The bootstrap 95% confidence intervals of several top-performing models overlapped substantially, indicating that the apparent differences among these classifiers should be interpreted cautiously. Therefore, the algorithm comparison was regarded as an exploratory analysis under a fixed preselected feature space rather than definitive evidence of algorithmic superiority.

**Figure 4:**
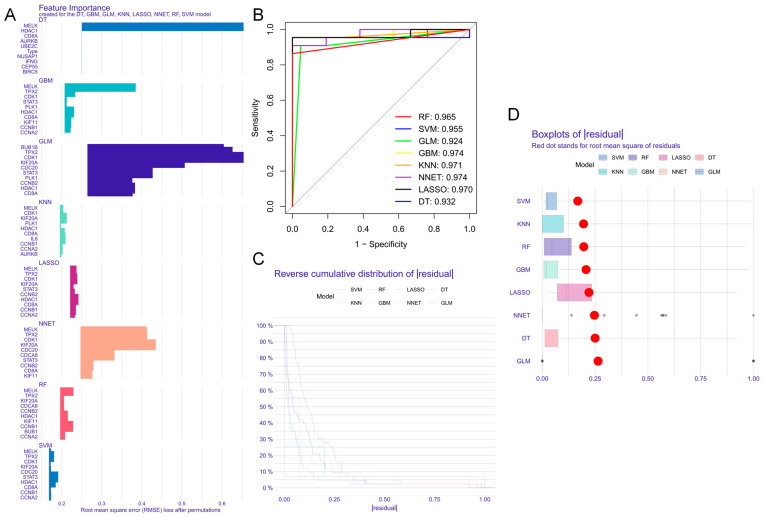
Exploratory comparison of eight machine learning algorithms on the preselected 25-gene feature set. (**A**) Feature importance bar plots for the eight algorithms. (**B**) ROC curve comparison. (**C**) Reverse cumulative distribution plot of residuals. (**D**) Residual boxplots; red dots indicate RMSE values, and grey dots represent outliers in the distribution of absolute residuals. Cross-validation AUCs with bootstrap 95% confidence intervals and standard classification metrics are summarized in Table 1.

Because several classifiers exhibited similar point-estimate AUCs with overlapping bootstrap confidence intervals, residual analysis was introduced as a complementary descriptive evaluation of prediction error distribution. The reverse cumulative distribution plot of residuals (Figure 4C) showed that GBM exhibited a relatively steep rising curve, suggesting that its prediction residuals tended to be concentrated in a lower range under the present fixed-feature analysis. The residual boxplot (Figure 4D) further suggested that GBM had a compact residual distribution and a low RMSE under the present analysis. However, because the algorithm comparison was not fully nested, these findings should be regarded as exploratory rather than definitive evidence of superior predictive stability. In contrast, although NNET matched GBM in AUC, its residual distribution was more dispersed, indicating slightly inferior stability. The feature importance bar plots across algorithms (Figure 4A) revealed pronounced differences in weight assignment to the 25 hub genes under different mathematical frameworks, further validating the necessity of multi-algorithm comparison, as a single algorithm may overlook or overestimate the diagnostic value of certain genes. Based on the joint descriptive evaluation of AUC, standard classification metrics, residual-based stability, and interpretability considerations within the preselected feature set, GBM was retained for downstream exploratory construction of the psoriasis molecular classification model. This selection should not be interpreted as definitive evidence that GBM is superior to the other algorithms under a fully unbiased nested resampling framework.

### 2.4. Explainability Analysis, Core Feature Gene Identification, and Model Validation

DALEX-based explainability analysis of the GBM model produced permutation-based feature importance scores for each hub gene. Based on the ranking, five candidate apoptosis-associated and proliferation-apoptosis-coupled feature genes were identified: *CCNB1* (Cyclin B1), *KIF11* (Kinesin Family Member 11), *HDAC1* (Histone Deacetylase 1), *TPX2* (Targeting Protein for Xklp2), and *MELK* (Maternal Embryonic Leucine Zipper Kinase). All five genes were retrieved from the GeneCards apoptosis-related gene set with relevance scores >2, suggesting a documented but broad association with apoptosis-related biological processes.

**Figure 5:**
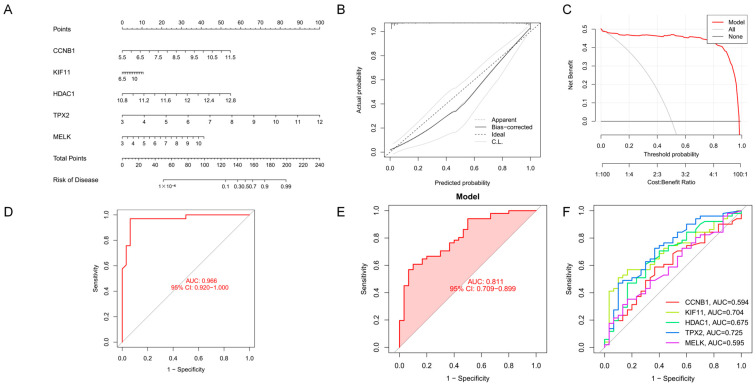
Construction and evaluation of the five-gene classification model. (**A**) Nomogram for quantitative psoriasis risk prediction. (**B**) Calibration curve. (**C**) Decision curve analysis. (**D**) ROC curve in the training cohort. (**E**) ROC curve in the external validation cohort. (**F**) Individual ROC curves for each of the five core feature genes.

Based on the expression levels of these five core genes, a nomogram style visualization of the five gene classification model was constructed (Figure 5A). Each gene’s expression level is mapped to a corresponding score, and summing the individual scores yields a total score that corresponds to an individual’s probability of psoriasis, thereby providing an interpretable visualization of the model-derived psoriasis probability [30]. In the training cohort, the calibration curve (Figure 5B) showed that the bias-corrected curve was close to the ideal diagonal, suggesting apparent agreement between predicted and observed probabilities within the model-development dataset. DCA (Figure 5C) was performed in the training cohort as an exploratory analysis. Because clinically established actionable threshold probabilities for transcriptomic testing in psoriasis are currently unavailable and DCA was not repeated in the external validation cohort, these results should not be interpreted as evidence of clinical utility. Internal ROC analysis in the training cohort (Figure 5D) yielded an AUC of 0.966 (95% CI: 0.920–1.000) for the combined five-gene model, indicating strong apparent discrimination within the model-development dataset.

To evaluate the model’s generalizability, the five-gene classification model was applied to the fully independent external validation cohort GSE55201 (*n* = 74). The results (Figure 5E) showed that the combined model achieved moderate cross-tissue validation performance in the external whole-blood dataset, with an AUC of 0.811 (95% CI: 0.709–0.899). The decrease in AUC from the training cohort to the external validation cohort suggests that the model’s performance is affected by cross-cohort heterogeneity and that the internal AUC should be interpreted as apparent performance rather than an unbiased estimate of generalizability. Because GSE55201 was independently preprocessed and was not used during feature selection or model training, this result reduces the risk of information leakage from the validation cohort. It should be noted that this external validation cohort contained baseline psoriasis blood samples and healthy-control blood samples; therefore, the AUC reflects the model’s ability to distinguish psoriasis patients from healthy controls in a blood-based transcriptomic cohort, rather than direct validation in psoriatic skin lesions or discrimination from clinically similar dermatoses. Single-gene ROC analysis (Figure 5F) indicated that *TPX2* (AUC = 0.725) and *KIF11* (AUC = 0.704) exhibited the highest individual classification performance; however, all single-gene AUC values were lower than that of the combined five-gene model, suggesting that the combined panel provided better classification performance than individual genes in the analyzed dataset. It should be noted that GSE55201 is a whole-blood transcriptomic dataset containing baseline psoriasis samples and healthy controls. Therefore, the external validation AUC reflects the model’s ability to distinguish psoriasis patients from healthy controls in a blood-based transcriptomic cohort, rather than its ability to distinguish psoriasis from healthy controls at the transcriptomic level or to differentiate psoriasis from clinically similar dermatoses. To further evaluate whether the proposed five-gene panel provided diagnostic information beyond established psoriasis-related transcriptomic signatures, we performed a head-to-head ROC comparison with signatures representing the IL-17/IL-23 axis, keratinocyte differentiation/S100 markers, and antimicrobial peptides. The results are summarized in Supplementary Tables S1 and S2.

## 3. Discussion

### 3.1. Summary of Key Findings

Psoriasis is characterized by substantial molecular and clinical heterogeneity, highlighting the need for objective molecular markers that can complement conventional morphological and histopathological assessment [31,32]. In the present study, however, the available training and validation datasets were designed to compare psoriatic lesional skin with normal skin. Therefore, the current findings should be interpreted as evidence supporting psoriasis-versus-normal molecular classification rather than direct evidence for differential diagnosis against clinically similar dermatoses. Current clinical practice relies on morphological examination and histopathological biopsy, both of which are influenced by physician experience, biopsy site, and disease stage [33]. Therefore, the development of an objective and molecularly grounded supplementary molecular classification framework is of considerable value.

This study systematically integrated apoptosis-related gene data with multi-cohort high-throughput transcriptomic data through a complete analytical pipeline encompassing apoptosis-related gene screening, PPI hub gene identification, horizontal comparison of eight algorithms, DALEX-based explainability analysis, nomogram construction, and independent external validation. Starting from 894 apoptosis-related DEGs at the intersection of 3707 DEGs and 4249 apoptosis-related genes, 25 hub genes were selected via PPI network analysis. Within this preselected feature set, eight machine-learning algorithms were compared in an exploratory non-nested framework. Bootstrap analysis showed overlapping 95% confidence intervals among several top-performing classifiers, indicating that the apparent performance differences should be interpreted cautiously. GBM was retained for downstream exploratory explainability analysis based on its overall descriptive performance and compatibility with DALEX-based interpretation, through which five core apoptosis-associated and proliferation-related feature genes—*CCNB1*, *KIF11*, *HDAC1*, *TPX2*, and *MELK*—were identified. The resulting five-gene nomogram achieved an AUC of 0.966 in the training cohort and 0.811 in the fully independent external validation cohort. Calibration and DCA were performed only in the training cohort and were therefore interpreted as exploratory analyses of apparent model behavior rather than evidence of clinical utility. Unlike previous studies that rely on generalized screening of all differentially expressed genes [34,35], the present study starts from the core pathological feature of psoriasis—the imbalance between apoptosis and proliferation—to construct a biologically guided classification model with clear thematic focus on apoptosis regulation.

### 3.2. Functional Context: Literature-Based Interpretation of the Five-Gene Proliferation–Apoptosis-Coupled Signature

The five identified genes should not be interpreted as canonical apoptosis-execution genes. Rather, they represent apoptosis-associated and proliferation-apoptosis-coupled regulators that may reflect the disturbed coupling between keratinocyte hyperproliferation and impaired apoptotic control in psoriasis. The five core feature genes identified by our pipeline are members of the GeneCards apoptosis-related gene set (relevance score >2), and previous studies have independently reported associations between these genes and broad apoptosis-related biological processes. CCNB1 and TPX2 are involved in G2/M checkpoint regulation and mitotic progression, processes that have been linked in previous studies to mitotic cell death and the p53–Bax apoptotic axis [36]; loss of proper mitotic control may trigger mitotic catastrophe, an apoptosis-related cell death modality. KIF11, as a core mitotic motor protein, has been reported to activate caspase-3/9 and induce apoptosis when inhibited, and its elevated expression has been associated with resistance to apoptotic signals in hyperproliferative cells [37]. HDAC1 has been reported to epigenetically regulate pro-apoptotic genes including *p21*, *Bax*, and *PUMA*, suggesting a potential role in modulating apoptotic execution [38]. MELK, a serine/threonine kinase of the AMPK family, has been reported to phosphorylate FoxO3a and suppress the transcriptional activation of pro-apoptotic *Bim* and *FasL* in specific biological contexts [39].

From a literature-based perspective, the five-gene signature may reflect two complementary facets of proliferation–apoptosis imbalance in psoriatic keratinocytes. *CCNB1*, *TPX2*, and *KIF11* together form a cell-cycle and mitotic-regulation component whose overexpression shortens the G2/M transition and thereby limits the window during which apoptotic surveillance can eliminate damaged or hyperproliferative cells [40,41]., a mechanism directly relevant to the aberrant keratinocyte turnover observed in psoriasis. *HDAC1* and *MELK* constitute a regulatory component related to apoptosis resistance and cell-cycle–apoptosis coupling: *HDAC1* has been repeatedly reported to be significantly upregulated in psoriatic lesions [42,43], silencing apoptotic effectors at the transcriptional level, while MELK blocks apoptotic signaling at the post-translational level [39]. HDAC1 can additionally influence the transcription of *CCNB1* through chromatin remodeling, and MELK kinase activity has been reported to modulate G2/M checkpoint function [44]. These observations suggest possible links between epigenetic regulation, kinase signaling, and cell-cycle–apoptosis coupling, but such links were inferred from prior literature rather than demonstrated experimentally in the present study. This multilayered coupling explains why the combined five-gene model achieved better classification performance than individual genes in the analyzed datasets: each single gene reflects only one facet of the apoptosis–proliferation imbalance, whereas the five-gene panel may capture complementary aspects of the psoriasis-associated transcriptomic state of psoriatic keratinocytes across epigenetic silencing, kinase signaling, and cell-cycle–apoptosis coupling. These mechanistic interpretations are based on literature synthesis and remain to be directly validated in psoriatic keratinocytes through future experimental studies [45].

It is important to acknowledge that four of the five genes in the final panel, namely CCNB1, KIF11, TPX2, and MELK, are primarily involved in mitotic progression, spindle assembly, and cell-cycle regulation, whereas *HDAC1* is an epigenetic regulator whose relationship with apoptosis is context-dependent and relatively indirect. Therefore, the five-gene panel should not be considered a purely apoptosis-associated signature. Instead, it likely captures the proliferation–apoptosis imbalance that characterizes psoriatic epidermis. This interpretation is biologically plausible because keratinocyte hyperproliferation and apoptosis resistance are tightly coupled pathological processes in psoriasis. Nevertheless, PPI-based hub-gene selection may preferentially identify highly connected cell-cycle genes, and future studies should compare this signature with panels derived from curated apoptosis pathways and canonical apoptosis effectors.

### 3.3. Methodological Considerations of the Explainable Machine Learning Framework

A methodological feature of this study is the combination of an exploratory comparison of eight mainstream machine learning algorithms with the DALEX explainability framework. This design helps reduce reliance on a single arbitrary classifier and improves model interpretability, although the algorithm comparison should be interpreted with caution because upstream feature selection was performed before cross-validation. On the one hand, multi-algorithm comparison based on a unified evaluation framework (AUC, standard classification metrics, and RMSE-based residual stability) provides a descriptive overview of classifier behavior within the preselected feature set. In this study, although GBM and NNET both achieved point-estimate AUC values of 0.974, GBM exhibited a relatively compact residual distribution under the present analysis. However, because the upstream feature selection was not nested within the cross-validation loop, this observation should be interpreted as exploratory and cannot establish definitive superiority of GBM over NNET. Similar multi-algorithm comparison frameworks have been successfully applied to ischemic stroke [19] and cancer target identification [18], supporting the scientific validity of this strategy.

On the other hand, the DALEX framework quantified the marginal contribution of each gene to model performance through permutation-based feature importance analysis. The results showed that *MELK* exhibited the highest dropout loss (0.384), followed by *TPX2* (0.234), *HDAC1* (0.230), *KIF11* (0.224), and *CCNB1* (0.222). Notably, the feature importance of *MELK* was substantially higher than that of the other four genes (all within the 0.22–0.23 range), suggesting that MELK may serve as a primary predictive driver in the model’s recognition of the psoriatic molecular phenotype, consistent with its established role in apoptosis suppression [39]; its specific functional role in psoriasis, however, requires dedicated experimental validation in future studies. This quantifiable and traceable feature contribution analysis improves the transparency of the model’s decision logic compared with conventional black-box classifiers. In summary, the explainable machine learning framework employed in this study offers three distinct methodological advantages: (i) the predictive decision process is transparent and traceable, with each feature gene’s contribution precisely quantified; (ii) the model output is more readily interpretable than conventional black-box outputs; and (iii) the quantified feature importance rankings indicate candidate molecules for subsequent functional validation and future functional investigation. The methodological framework established here—disease-specific gene set screening, multi-cohort transcriptomic integration, multi-algorithm comparison, explainability-based feature extraction, and nomogram-based translation—is modular and may be transferable to other immune-mediated inflammatory diseases [46], although such extensions require rigorous independent validation in each specific disease context.

Clinical positioning of the model. We emphasize that the current model should not be interpreted as a validated differential-diagnostic tool for distinguishing psoriasis from clinically or histologically similar skin diseases. The independent external validation cohort used in this study consisted of baseline psoriasis blood samples and healthy-control blood samples; thus, it evaluates psoriasis-versus-normal classification rather than discrimination between psoriasis and disease mimics such as eczema, seborrheic dermatitis, lichen planus, or cutaneous T-cell lymphoma. The appropriate positioning of the present model is therefore a research-oriented apoptosis-associated and proliferation–apoptosis-coupled molecular classification framework. Its potential value lies in identifying biologically interpretable candidate biomarkers and providing a basis for future mechanistic and diagnostic studies. Before any claim regarding differential diagnosis can be made, the five-gene model must be tested in independent disease-control cohorts including inflammatory and neoplastic skin disorders that clinically resemble psoriasis. Because the current model is based on skin tissue transcriptomic data, any translation to peripheral blood assays would also require dedicated validation in paired skin–blood cohorts.

### 3.4. Study Limitations

Several important limitations of the present study warrant explicit discussion.

First, although the model was externally validated in an independent GEO cohort, the validation dataset included only baseline psoriasis blood samples and healthy-control blood samples. Therefore, the validation design does not directly address the clinically more challenging task of distinguishing psoriasis from disease mimics, such as eczema, atopic dermatitis, seborrheic dermatitis, lichen planus, or cutaneous T-cell lymphoma. The AUC of 0.811 in the external cohort should be interpreted as evidence of psoriasis-versus-normal classification performance rather than differential-diagnostic performance. Future studies should validate the five-gene model in independent disease-control datasets and prospective cohorts containing clinically similar inflammatory and neoplastic skin diseases.

Second, feature selection procedures, including DEG screening, apoptosis-gene intersection, PPI network construction, and hub-gene identification, were performed on the complete training cohort prior to cross-validation. This design may lead to optimistically biased cross-validation AUCs and may also affect the apparent ranking among algorithms. The decrease in AUC from the training cohort (0.966) to the external validation cohort (0.811) further suggests that the internal performance estimate may overstate generalizability. This discrepancy may reflect multiple sources of heterogeneity between the model-development and validation datasets, including differences in cohort composition, sample source, preprocessing history, clinical background, and residual batch effects that cannot be fully eliminated by annotation harmonization alone. Therefore, the eight-algorithm comparison should be interpreted as an exploratory descriptive analysis within a preselected feature set, and the external AUC of 0.811 on GSE55201, which was not used at any stage of feature selection or model training, should be regarded as the primary evidence for model generalizability. Future studies should adopt fully nested resampling procedures and validate the model in larger, independently collected cohorts.

Third, feature selection procedures (DEG screening, apoptosis gene intersection, PPI network, and hub gene identification) were performed on the complete training cohort prior to cross-validation. This common practice in gene-signature studies may lead to optimistically biased cross-validation estimates; we therefore regard the external validation AUC of 0.811 on GSE55201 (not used at any stage of feature selection) as the primary evidence for model generalizability. Fully nested feature selection–training loops were not adopted due to limited per-dataset sample sizes and represent a meaningful direction for future work.

Fourth, both cohorts were derived from predominantly Caucasian populations [47], and applicability to Asian, African, and other populations requires evaluation in ethnically diverse cohorts, particularly given known population-level differences in psoriasis susceptibility loci.

Fifth, the model was developed on skin tissue transcriptomes; translation to peripheral blood biomarker panels would require verification of expression consistency in paired skin–blood samples [48], which is a prerequisite for any minimally invasive diagnostic application.

Sixth, prospective clinical cohort validation is currently lacking. Calibration and DCA were performed only in the training cohort and should be interpreted as exploratory descriptive analyses under retrospective public-data conditions rather than evidence of real-world clinical utility.

Seventh, apoptosis-related genes were retrieved from GeneCards using a relevance score threshold >2, which yielded a broad gene set and may have included genes only indirectly associated with programmed cell death. Moreover, the final five-gene panel was enriched for cell-cycle and mitotic regulators, including *CCNB1*, *KIF11*, *TPX2*, and *MELK*. This raises the possibility that the model primarily reflects keratinocyte hyperproliferation and proliferation–apoptosis coupling rather than canonical apoptosis-effector activity. Future studies should repeat the analysis using more curated apoptosis resources, including GO:0006915, Reactome apoptosis pathways, KEGG hsa04210, and MSigDB HALLMARK_APOPTOSIS, and compare the model with panels composed of canonical apoptosis effectors such as *BAX*, *BCL2*, *FAS*, *FASLG*, *CASP3*, *CASP8*, *CASP9*, *TNFRSF10A*, and *TNFRSF10B*.

Another limitation is that the present study did not perform a head-to-head comparison between the five-gene panel and established psoriasis-associated transcriptomic signatures, including IL-17/IL-23 axis genes, keratinocyte differentiation markers, and antimicrobial peptides. Therefore, whether the proposed panel provides incremental diagnostic value beyond canonical inflammatory or keratinocyte-differentiation signatures remains unclear. Future studies should compare these signatures directly using ROC analysis in both training and independent validation cohorts.

## 4. Materials and Methods

### 4.1. Data Acquisition and Preprocessing

Transcriptomic gene expression data were obtained from the GEO database (https://www.ncbi.nlm.nih.gov/geo/, accessed on 16 March 2026) [17]. Two independent skin transcriptomic datasets based on the GPL570 platform (Affymetrix Human Genome U133 Plus 2.0 Array) were downloaded: GSE30999 (85 psoriatic lesional skin samples and 85 normal skin samples) and GSE53552 (50 psoriatic lesional skin samples and 49 normal skin samples). These two datasets were merged to form a skin model-development cohort of 269 samples to increase sample size and enhance statistical power.

GSE55201 was used as an independent external validation cohort. This dataset was also generated on the GPL570 platform and contains whole-blood transcriptomic profiles from healthy controls and patients with psoriasis before and after anti-IL-17 treatment. The original dataset contained 81 samples, including 30 healthy controls, 44 baseline psoriasis samples, and 7 post-treatment samples. Because post-treatment samples may exhibit treatment-induced transcriptional changes, the 7 treated samples were excluded. Therefore, the final external validation cohort consisted of 74 whole-blood samples, including 30 healthy controls and 44 baseline psoriasis samples.

Probe IDs in each dataset were mapped to gene symbols using the corresponding GPL570 annotation file, and when multiple probes mapped to the same gene, the mean expression value was used. The whole-blood external validation cohort was preprocessed independently from the skin model-development cohort. No co-normalization, ComBat adjustment, or cross-cohort batch correction was performed between the skin model-development cohort and the whole-blood external validation cohort. The batch-effect correction using the limma removeBatchEffect function was applied only to the merged skin model-development cohort composed of GSE30999 and GSE53552. GSE55201 was not used during DEG screening, apoptosis-gene intersection, PPI network construction, hub-gene identification, model training, hyperparameter tuning, DALEX feature selection, or nomogram construction. The external validation cohort was used only after the final five-gene model had been fixed, thereby minimizing information leakage from the validation set into preprocessing, feature selection, or model development.

Because GSE30999 and GSE53552 originated from different experimental batches, the removeBatchEffect function in the limma package (version 3.66.0) [49] of R (version 4.3.0) was applied to the merged expression matrix for batch-effect correction. Principal component analysis (PCA) was used to visualize sample distribution before and after correction, with successful correction defined as uniform intermixing of samples from both datasets in PCA space [50]. It should be noted that the independent external validation was performed in a whole-blood cohort rather than an independent skin tissue cohort; therefore, the external validation results should be interpreted as cross-cohort and cross-tissue evaluation of psoriasis-versus-healthy-control discrimination.

### 4.2. Differential Expression Analysis and Apoptosis-Related Gene Intersection Screening

The limma package (version 3.66.0) [49] was used to perform differential expression analysis on the batch-corrected integrated training cohort. After constructing the group design matrix, linear model fitting and empirical Bayes methods were applied to compute log fold changes and adjusted *p*-values for each gene. The Benjamini–Hochberg method was used to control the false discovery rate (FDR). DEGs were identified using the criteria |log2FC| > 0.5 and FDR < 0.05, and results were visualized using volcano plots and hierarchical clustering heatmaps.

Apoptosis-related genes were retrieved from the GeneCards database (https://www.genecards.org) [51] using “Apoptosis” as the search keyword, with a relevance score threshold >2 to select moderately to highly apoptosis-associated genes. The GeneCards relevance score is a weighted score that integrates multiple evidence sources including GO annotations, KEGG pathways, literature curation, and functional databases, ensuring that only genes with substantial apoptosis-related functional evidence were retained. The intersection between DEGs and the apoptosis-related gene set yielded the apoptosis-related DEGs, which were visualized using a Venn diagram.

### 4.3. PPI Network Construction and Hub Gene Analysis

The apoptosis-related DEGs were submitted to the STRING database (https://cn.string-db.org/; species: Homo sapiens) with the highest confidence threshold (confidence = 0.9) to construct a protein–protein interaction (PPI) network [52]. The resulting network was imported into Cytoscape software (version 3.10.4) [53] for visualization and topological analysis. The cytoHubba plugin [54] was employed to evaluate node importance using three complementary topological algorithms—Degree, Maximal Clique Centrality (MCC), and Closeness—and the top 25 genes ranked by connectivity were selected as hub genes.

Pearson correlation analysis was performed on the 25 hub genes to explore transcriptional co-regulatory relationships (significance threshold *p* < 0.05), with results visualized using a correlation heatmap and chord diagram. Chromosomal coordinate information for each hub gene was obtained from the GeneCards database, and a Circos plot was generated using the circlize R package [55] based on the hg38 human genome assembly to display the genomic distribution of hub genes.

### 4.4. Machine Learning Modeling and Explainability Analysis

Expression data of the hub genes were extracted from the integrated training cohort, and predictive models were constructed using the caret R package [56]. The following eight machine learning algorithms were systematically trained: Random Forest (RF), Support Vector Machine (SVM), Generalized Linear Model (GLM), Gradient Boosting Machine (GBM) [57], K-Nearest Neighbors (KNN), Neural Network (NNET), Least Absolute Shrinkage and Selection Operator (LASSO) [20], and Decision Tree (DT). All eight algorithms were trained using the same preselected 25 hub genes as input features. Thus, the comparison was designed to assess classifier performance within a common biologically guided feature space, rather than to compare independent modeling pipelines with separate algorithm-specific feature-selection steps.

These eight algorithms encompass the major mathematical paradigms of contemporary machine learning. RF reduces overfitting risk by aggregating predictions from numerous independent decision trees via majority voting [21]. SVM identifies an optimal separating hyperplane in a high-dimensional feature space and is well suited for classification tasks with small sample sizes and high dimensionality [22]. GLM extends linear regression to binary classification, offering simple model structure and directly interpretable coefficients, and commonly serves as a baseline model. GBM sequentially adds weak learners that progressively fit the residuals of the previous iteration, generally excelling on structured data [57]. KNN classifies samples via majority voting among their nearest neighbors without explicit model training but is sensitive to feature scaling. NNET models complex mappings through multi-layer nonlinear transformations, offering strong fitting capacity but lower interpretability [58]. LASSO introduces an L1 regularization penalty into linear regression, shrinking unimportant feature coefficients to zero for automatic variable selection [20]. DT partitions feature space recursively into interpretable decision rules but is prone to overfitting when used alone [59]. Comparing these eight algorithms spans ensemble learning, kernel methods, linear models, gradient optimization, instance-based learning, neural networks, regularized regression, and rule-based learning, thereby minimizing single-algorithm bias.

Prior to model training, the expression matrix of the selected hub genes was transposed so that samples were arranged in rows and genes in columns, and the sample class label was extracted from the sample names. A fixed random seed was used to improve reproducibility (set.seed(123)). The integrated training cohort was divided into an internal training set and an internal testing set using the createDataPartition function in the caret package, with 80% of samples assigned to the internal training set and 20% assigned to the internal testing set. Because createDataPartition was applied to the outcome variable, stratified sampling was used to preserve the case–control distribution across the two subsets.

Model training was performed in the internal training set using the caret package. The resampling strategy was repeated five-fold cross-validation implemented by trainControl (method = “repeatedcv”, number = 5, savePredictions = TRUE), with saved resampling predictions. The number of repeats was not manually modified beyond the caret setting used in the retained analysis script. No explicit oversampling, undersampling, synthetic sampling, or class-weight adjustment was applied, because the case–control distribution was relatively balanced in the model-development dataset.

Eight machine learning algorithms were trained using the same preselected 25 hub genes as input features: Random Forest (RF), Support Vector Machine with radial kernel (SVM), Generalized Linear Model (GLM), Gradient Boosting Machine (GBM), K-Nearest Neighbors (KNN), Neural Network (NNET), Least Absolute Shrinkage and Selection Operator (LASSO), and Decision Tree (DT). Thus, the algorithm comparison was designed to assess classifier performance within a common biologically guided feature space rather than to compare fully independent end-to-end modeling pipelines with separate algorithm-specific feature-selection procedures.

Model discrimination was evaluated in the internal testing set using predicted class probabilities, ROC curves, and AUC values calculated by the pROC package. Standard classification metrics, including Accuracy, Precision, Recall, and F1-score, were calculated at the default probability threshold of 0.5. DALEX was used to evaluate residual-based model performance and permutation-based feature importance. The algorithm used for downstream exploratory model construction was selected according to the joint descriptive criterion of point-estimate AUC, standard classification metrics, and residual distribution within the preselected 25-gene feature set. Because upstream feature selection was performed before cross-validation, this selection was not intended to represent an unbiased nested comparison of algorithmic superiority.

Model evaluation was conducted along three complementary dimensions. First, discriminatory ability was assessed via AUC based on receiver operating characteristic (ROC) curves [60]. Second, to complement AUC with conventional binary classification metrics, Accuracy, Precision, Recall, and F1-score were computed at the default 0.5 probability threshold. Third, predictive stability was evaluated using residual boxplots and reverse cumulative distribution plots of RMSE. In this study, RMSE is not intended as a primary classification metric, but rather as a complementary stability indicator derived from the DALEX framework, reflecting the consistency of predicted probabilities across samples and providing a tiebreaker when AUC values are identical. The algorithm used for downstream exploratory model construction was selected according to the joint descriptive criterion of point-estimate AUC, standard classification metrics, and residual distribution within the preselected 25-gene feature set. Because upstream feature selection was performed before cross-validation, this selection was not intended to represent an unbiased nested comparison of algorithmic superiority.

It should be noted that the feature selection procedures used in this study (DEG screening, apoptosis-related gene intersection, PPI network construction, and hub gene identification) were performed on the complete integrated training cohort prior to cross-validation. As a result, the cross-validation AUC reported here primarily reflects the relative performance of different algorithms given the same pre-selected feature set, rather than a fully unbiased estimate of out-of-sample generalization. The independent external validation cohort (GSE55201, *n* = 74), which was not used at any stage of feature selection or model training, therefore serves as the primary evidence for model generalizability, and the cross-validation results should be interpreted accordingly.

The DALEX R package [29] was then applied to the optimal model for explainability analysis. DALEX provides a unified “explainer” interface and computes the marginal contribution of each gene to model performance (dropout loss) using a permutation-based feature importance algorithm. The top five genes ranked by DALEX feature importance were selected to construct a parsimonious exploratory molecular classification panel. This panel size was chosen to improve interpretability and reduce model complexity, but it was not intended to represent an optimized cutoff across all possible panel sizes.

### 4.5. Nomogram Construction and External Validation

A risk-score visualization for quantitative psoriasis risk prediction was constructed using the rms R package [61] based on the expression levels of the five diagnostic feature genes. Calibration curves were plotted in the training cohort to evaluate apparent agreement between predicted and observed probabilities. Decision curve analysis (DCA) [62] was also performed in the training cohort as an exploratory analysis. Because clinically established actionable threshold probabilities for transcriptomic testing in psoriasis are unavailable, no specific threshold range was interpreted as clinically actionable. Finally, the five-gene classification model was applied to the independent external validation cohort GSE55201, with ROC curves plotted and AUC values calculated to evaluate both the combined model and the diagnostic performance of each individual gene. It should be emphasized that both the nomogram calibration and DCA were performed on the training cohort, and therefore represent a theoretical estimate of net benefit under retrospective public-data conditions rather than a demonstration of real-world clinical utility.

### 4.6. Statistical Analysis

All data processing and statistical analyses were performed using R software (version 4.3.0). Between-group comparisons were conducted using the Wilcoxon rank-sum test, and correlation analyses were performed using the Pearson correlation coefficient. All statistical tests were two-tailed, and *p* < 0.05 was considered statistically significant.

## 5. Conclusions

By integrating multi-cohort psoriasis transcriptomic data from the GEO database with apoptosis-associated gene information from the GeneCards database, this study screened apoptosis-associated differentially expressed genes and constructed an exploratory five-gene molecular classification model based on *CCNB1*, *KIF11*, *HDAC1*, *TPX2*, and *MELK* through PPI network analysis, comparison of eight machine learning algorithms, and explainability-based feature importance analysis. Using GBM for downstream exploratory model construction, the five-gene model demonstrated favorable performance in the internal training set (AUC = 0.966) and maintained moderate discriminatory ability in the fully independent external validation set (AUC = 0.811). The five feature genes are associated with apoptosis-related biological processes but are mainly linked to cell-cycle regulation, mitotic progression, epigenetic control, and kinase signaling; therefore, the panel is best interpreted as an apoptosis-associated and proliferation–apoptosis-coupled transcriptomic signature. The model is positioned as a research-oriented apoptosis-related molecular classification framework for distinguishing psoriasis from healthy controls at the transcriptomic level, rather than as a replacement for histopathological biopsy or a screening device ready for population-level deployment. Its differential-diagnostic value for early or atypical psoriasis against clinically similar dermatoses remains unproven and requires validation in independent disease-control cohorts. The five feature genes represent candidate targets for future experimental validation and future functional validation. Beyond the specific biological findings, this study provides a potentially transferable methodological framework for applying explainable artificial intelligence to transcriptomic biomarker discovery in dermatological and other complex inflammatory diseases.

## Figures and Tables

**Table 1:** Cross-validation performance of eight machine-learning algorithms on the preselected 25-gene feature set.

Rank	Model	CV AUC	Bootstrap 95% CI	Accuracy	Precision	Recall	F1-Score
1	KNN	0.978	0.955–0.994	0.909	0.909	0.909	0.909
2	RF	0.974	0.948–0.991	0.932	0.952	0.909	0.930
3	SVM	0.971	0.945–0.992	0.932	0.952	0.909	0.930
4	LASSO	0.969	0.940–0.993	0.955	0.955	0.955	0.955
5	NNET	0.962	0.936–0.986	0.955	0.955	0.955	0.955
6	GBM	0.962	0.931–0.986	0.932	0.952	0.909	0.930
7	GLM	0.933	0.893–0.966	0.932	0.913	0.955	0.933
8	DT	0.890	0.849–0.931	0.864	0.864	0.864	0.864

## Data Availability

The transcriptomic datasets analyzed in this study are publicly available from the NCBI GEO repository (https://www.ncbi.nlm.nih.gov/geo/, accessed on 16 March 2026) under accession numbers GSE30999, GSE53552, and GSE55201. Apoptosis-related gene data are available from the GeneCards database (https://www.genecards.org). The customized R scripts and analytical workflow used in this study have been deposited in a public GitHub repository (https://github.com/liuxinhao872-create/IJMS-Psoriasis-Code, accessed on 15 May 2026) and are permanently archived in Zenodo with the DOI: https://doi.org/10.5281/zenodo.20234695 (accessed on 15 May 2026).

## Supplementary Materials

The following supporting information can be downloaded at https://www.mdpi.com/article/10.3390/ijms27125441/s1.
