# Supplementary figures and images for: An Exploratory Transcriptomic Classification Model for Psoriasis Based on Apoptosis-Associated and Proliferation–Apoptosis-Coupled Genes Using Explainable Machine Learning
Source: Int J Mol Sci. 2026 Jun 16;27(12):5441. doi: 10.3390/ijms27125441 (PMC13299817; doi:10.3390/ijms27125441)

Sensitivity analysis across panel sizes

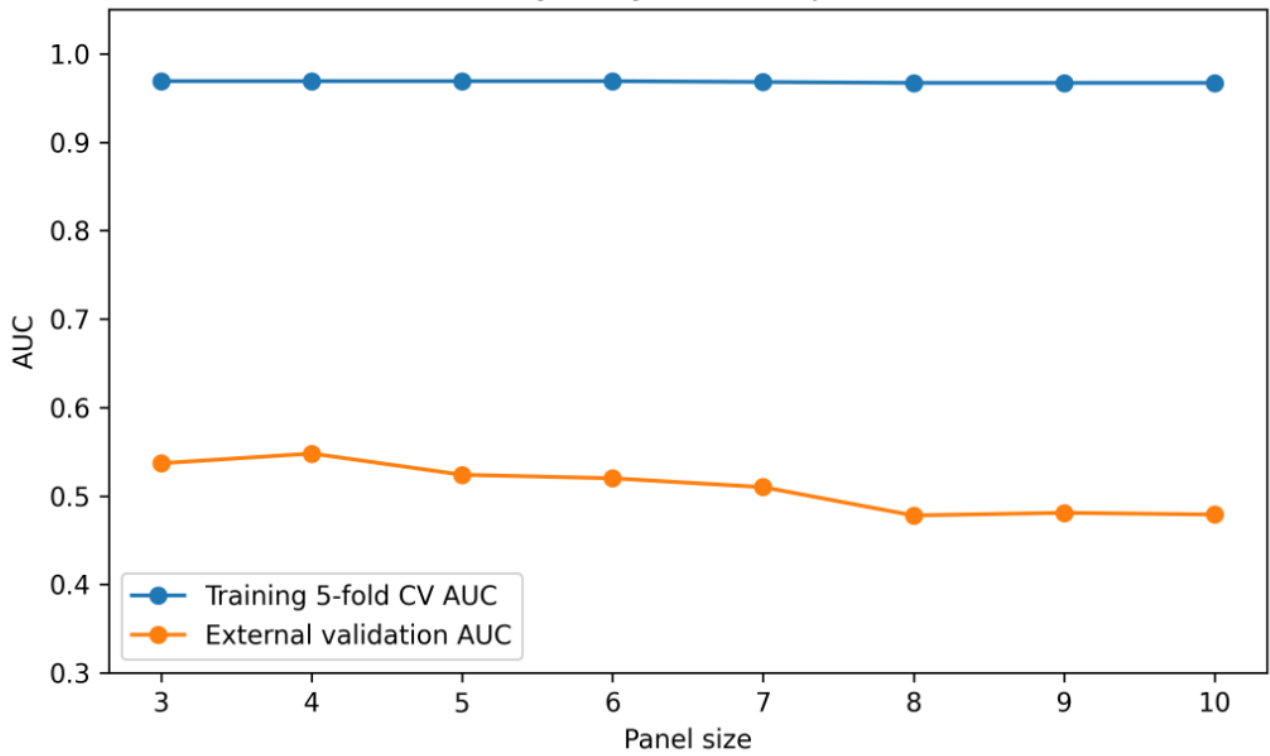

Sensitivity analysis across hub-set sizes

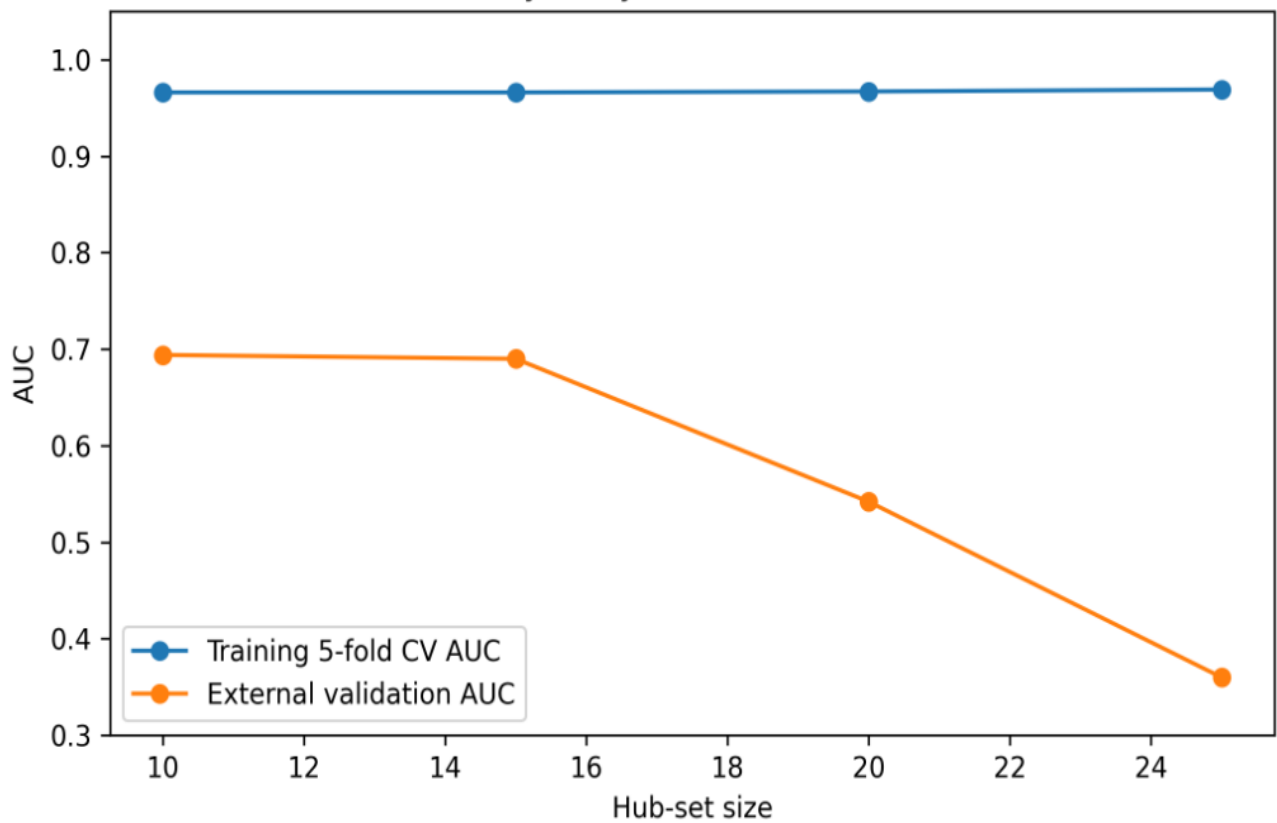

Supplement: Supplementary file 1 [file ijms-27-05441-s001.zip › Supplementary Figure S1.pdf]
